# Exogenous melatonin alleviates post-flowering natural high-temperature stress in maize (*Zea mays* L.) by promoting photosynthesis and antioxidant defense responses

**DOI:** 10.3389/fpls.2026.1810561

**Published:** 2026-04-28

**Authors:** Feiyan Ju, Chunhua Gao, Fengtao Zhao, Weilin Kong, Zongxin Li, Haijun Zhao, Kaichang Liu, Ping Liu

**Affiliations:** 1 Institute of Industrial Crops, Shandong Academy of Agricultural Sciences, Jinan, China; 2 State Key Laboratory of Nutrient Use and Management, Key Laboratory of Agro-Environment in Huang-Huai-Hai Plain, Institute of Agricultural Resources and Environment, Shandong Academy of Agricultural Sciences, Jinan, China; 3 Shandong Academy of Agricultural Sciences, Jinan, China

**Keywords:** heat stress, maize, melatonin, photosynthesis, reactive oxygen species scavenging

## Abstract

**Introduction:**

High-temperature stress severely restricts the growth, development and yield formation of summer maize. Foliar application of exogenous melatonin has been widely demonstrated to enhance stress tolerance and alleviate abiotic stress in crops. However, the physiological and molecular mechanisms underlying melatonin-mediated thermotolerance in summer maize under post-flowering high-temperature stress remain unclear.

**Methods:**

In this study, field experiments were conducted using foliar application of melatonin at different concentrations on maize. By integrating agronomic, physiological and transcriptomic approaches, we systematically investigated the mechanisms by which melatonin improves the adaptation of summer maize to post-flowering high-temperature stress.

**Results and Discussion:**

The results showed that exogenous melatonin increased the net photosynthetic rate of ear leaves and maintained high photosynthetic capacity. Melatonin treatment also significantly increased the activities of key antioxidant enzymes and the contents of antioxidant substances in the maize ear leaves, thereby enhancing the antioxidant defense capacity of the plant. Transcriptomic analysis further revealed that melatonin upregulated the expression of key genes involed in the photosynthetic and antioxidant defense systems of maize. In summary, exogenous melatonin application improved the resistance of summer maize to natural post-flowering high-temperature stress by optimizing photosynthetic function and reinforcing the antioxidant defense system, effectively reducing the damage of high-temperature to maize growth, biomass accumulation and grain yield. The 150 µmol L-1 melatonin treatment exerted the optimal mitigating effect under field conditions. These findings help to elucidate the key mechanisms by which exogenous melatonin enhances thermotolerance in maize under post-flowering high-temperature stress, and provide a theoretical basis for research on of maize stress resistance, disaster reduction, yield improvement, and efficient chemical regulation strategies.

## 1 Introduction

Maize is a major food, economic and feed crop worldwide. In recent years, high-temperature weather has occurred with earlier onset, higher frequency and greater severity in many regions across the globe, and the warming trend has been continuously intensifying, which severely restricts the growth, development, and yield formation of maize. Summer maize in the Huang-Huai-Hai region of China is frequently subjected to heat stress, with temperatures exceeding 35 °C and even reaching 40 °C in some areas (Wang et al., 2018). Although maize is a thermophilic crop, its optimal temperature requirements vary among different growth stages. In general, the reproductive growth stage of maize is more sensitive to heat stress than the vegetative growth stage. Sustained high temperatures above 35 °C seriously affect the differentiation of maize tassels, leading to shortened main rachis, reduced branch number, disrupted flowering and pollen shedding, as well as decreased pollen viability, thereby impairing pollination and fertilization, and ultimately resulting in reduced seed-setting rate and grain yield in maize (McNellie et al., 2018). Previous studies have indicated that for summer maize during the flowering stage, the critical threshold for heat damage is an average daily temperature above 28 °C with three consecutive days of maximum temperature exceeding 32 °C, while the significant threshold for heat damage is two consecutive days with maximum temperature exceeding 35 °C (Deryng et al., 2014; Li et al., 2022a). The frequent occurrence of extreme heatwaves worldwide poses a serious threat to global food security (Perkins-Kirkpatrick et al., 2024). Therefore, exploring effective strategies for disaster mitigation, yield stabilization, cultivation improvement and stress prevention, as well as enhancing the thermotolerance of maize, is of great significance for ensuring high and stable production of maize.

Plant growth regulators (PGRs) effectively regulate plant growth and development by enhancing stress resistance, increasing yield, and improving plant architecture. The application of exogenous PGRs to improve crop thermotolerance is considered a simple and efficient cultivation strategy for yield enhancement, and has been validated in wheat (Chowdhury et al., 2020), rice (Li et al., 2020b), and horticultural crops (Gao et al., 2024). Melatonin (MT) is an indole derivative of tryptophan that enhances plant antioxidant capacity, maintains cell membrane stability, inhibits chlorophyll decomposition, regulates stomatal movement and reduces abscisic acid (ABA) content (Arnao and Josefa, 2007). Recent studies have shown that melatonin improves crop adaptability to adverse environmental stresses and alleviates the damage caused by various abiotic stresses (Posmyk et al., 2009; Xia et al., 2020). Melatonin has been reported to significantly enhance heat tolerance in Arabidopsis and tomato pollen by activating protein and enzyme pathways, effectively protecting plant and pollen activity (Shi et al., 2015; Qi et al., 2018). Furthermore, melatonin enhances antioxidant enzyme activities, promotes the accumulation of osmoticregulatory substances, reduces the reactive oxygen species (ROS) accumulation and alleviates oxidative damage in plants (Manchester et al., 2015; Dai et al., 2020). Yan et al. (2021) reported that exogenous melatonin enhanced photosynthetic efficiency by promoting the expression of antioxidant-related genes and increasing the activities of ROS-scavenging enzymes, thereby alleviating salt stress in crops. Similarly, Zhang et al. (2021a) demonstrated that melatonin enhanced antioxidant system capacity and soluble sugar accumulation by promoting K^+^ influx and Na^+^ efflux in beet leaves, thus improving salt tolerance. These results are consistent with findings by Antoniou et al. (2017) melatonin-mediated regulation of alfalfa growth under drought stress. Melatonin mitigates heat-induced damage by activating antioxidant mechanism, increasing superoxide dismutase (SOD) activity and ensuring normal survival of plant under high-temperature (Buttar et al., 2020). Jahan et al. (2021a) demonstrated that melatonin promoted CO_2_ assimilation, enhanced the activities of Rubisco and Fructose-1, 6-bisphosphatase (FBPase), and upregulated photosynthesis-related genes expression in tomato seedlings under high-temperature stress. Zhang et al. (2017) found that melatonin increased endogenous melatonin and cytokinins contents by significantly reducing ABA levels in wheatgrass leaves. Li et al. (2019) proposed that foliar application of 70 μmol L^-1^ melatonin reduced malondialdehyde (MDA) accumulation by enhancing peroxidase (POD) and glutathione reductase (GR) activities in leaves, significantly improving the survival rate of maize seedlings under high-temperature stress and alleviating heat damage. Collectively, these findings reveal the regulatory roles of melatonin in crop adaptive responses and also provide new insights into the use of exogenous melatonin as a protectant to alleviate abiotic stress in crops.

Extreme high temperatures after flowering severely restrict maize production (Cicchino et al., 2010; Hawkins et al., 2013; Li et al., 2022c). Although previous studies have demonstrated the regulatory roles of melatonin in crop growth, development and abiotic stress tolerance (Zhang et al., 2017; Jahan et al., 2021a; Teng et al., 2024; An et al., 2025), the specific mechanism underlying melatonin-mediated alleviation of post-flowering high-temperature stress in maize remains poorly understood. This study aimed to investigate the mitigating effect of exogenous melatonin on natural post-flowering high-temperature stress in maize, and to systematically evaluate the regulatory mechanisms on maize growth performance, photosynthetic function and antioxidant defense capacity under heat stress. These findings will help to elucidate the physiological and molecular mechanisms by which exogenous melatonin mediates maize tolerance to post-flowering high-temperature stress, and provide important theoretical guidance for research on the heat resistance mechanisms of maize.

## 2 Materials and methods

### 2.1 Plant material and growing conditions

**Figure 1:**
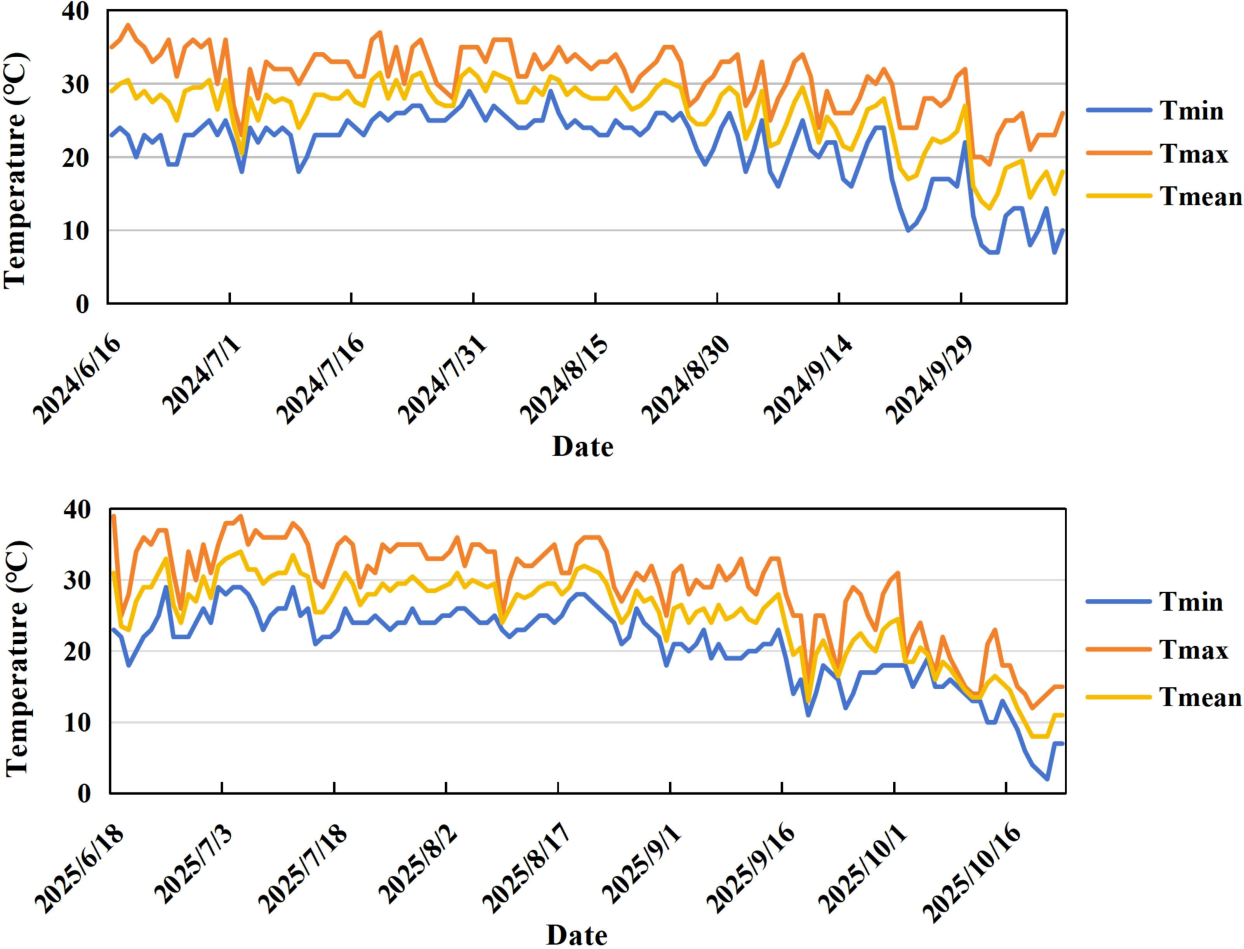
Temperature dynamics during the maize growing season in 2024 and 2025.

The experiment was conducted during the summer maize growing seasons of 2024 and 2025 at the Ruifeng Farmers ‘Professional Cooperative in Huantai County, Zibo City, Shandong Province (36°59’20.88” N, 118°067’27.21” E). This region has a warm temperate continental monsoon climate, with an annual mean sunshine duration of 2832 hours, an annual average temperature of 12.5-14.5 °C, and an annual average precipitation of 586.4 mm. The maize hybrid ‘MY73’ was used as the experimental material. This variety has a growth period of 101 days. It is characterized by purple seedling sheaths, green anthers and a compact plant type. The plant height is 238 cm, ear height is 94 cm, and the number of leaves at maturity is 20. It exhibits good stem elasticity, a well-developed and deep root system, and low plant gravity center, conferring strong lodging resistance. The ear is cylindrical with a white ear axis and yellow kernels. This hybrid shows high yield potential and excellent disease resistance, and is suitable for widespread planting in the summer maize region of the Huang-Huai-Hai Plain, China. Dynamic changes in temperature during the maize growing seasons in 2024 and 2025 are presented in **Figure 1**. Detailed temperature data are presented in **Supplementary Table 1**.

### 2.2 Experimental design

Maize was planted in alternating wide and narrow rows (80 cm - 50 cm - 80 cm) at a planting density of 82 500 plants hm^-2^. Each plot covered an area of 40 m² (8 m×5 m). Five treatments were arranged in the field experiment: (1) Control (CK): distilled water; (2) MT50: 50 µmol L^-1^ melatonin; (3) MT100: 100 µmol L^-1^ melatonin; (4) MT150: 150 µmol L^-1^ melatonin; (5) MT200: 200 µmol L^-1^ melatonin. Foliar spraying was applied at the tasseling stage (August 6, 2024 and August 5, 2025). The solution was evenly sprayed onto both adaxial and abaxial leaf surfaces using a backpack sprayer to ensure full leaf wetting. Spraying was conducted every 3 days for a total of 3 applications, with a spray volume of 750 kg hm^-2^. A randomized complete block design with three replicates was used in this experiment. All other field management practices followed local conventional cultivation measures. The location map of the experimental site and field planting layout are presented in **Supplementary Figure 1**.

### 2.3 Measurement of yield and plant growth traits

According to Geng et al. (2019), all maize plants in each plot were harvested at physiological maturity, and the fresh grain yield and harvested ear number were recorded. Grain yield per hectare was calculated accordingly. Ten ears with average representative ear weight were selected from each plot to determine ear fresh weight and ear dry weight. Ear length was measured using a ruler, while ear diameter and cob diameter were measured using a vernier caliper. Ear row number and kernels per row were also recorded. After threshing, grain fresh weight and hundred-grain weight were determined for each treatment with three replications. At harvest, three plants with uniform growth were sampled from each experimental plot. Plant height and ear height were measured using a ruler, and stem diameter was measured using a vernier caliper. Each treatment was performed in triplicate.

### 2.4 Determination of dry matter accumulation

At harvest, three plants with uniform growth were sampled from each plot. Plants were separated into four components: leaves, stems, bracts, and ears. All samples were oven-dried at 105 °C for 30 minutes, then dried to a constant weight at 65 °C. Subsequently, the dry matter weight of each organ was measured (Fu et al., 2023). The regulatory effects of exogenous melatonin at different concentrations on dry matter production and its partitioning among different organs were evaluated. Each treatment was performed in triplicate.

### 2.5 Determination of leaf gas exchange parameters

On day 14 after maize flowering, gas exchange parameters of maize ear leaves were measured using the LI-6400 (LI-COR Inc., USA) portable photosynthesis measurement system. All measurements were performed between 9:30 and 11:00 a.m. under stable natural light conditions. In each experimental plot, three uniformly growing and healthy maize plants without mechanical damage or disease symptoms were randomly selected. The net photosynthetic rate (*Pn*), stomatal conductance (*Gs*), intercellular CO_2_ concentration (*Ci*), and transpiration rate (*Tr*) were determined following the protocols described by Zhang et al. (2022). During measurement, only the middle portion of fully expanded ear leaves was measured while avoiding main leaf veins. Each treatment included three independent biological replicates to ensure data reliability.

### 2.6 Determination of antioxidant defense system indicators

On day 14 after maize flowering, three uniformly growing and healthy plants were randomly selected from each experimental plot. Maize ear leaves were sampled and immediately frozen in liquid nitrogen for cryopreservation, and the samples were used to determine the activities of antioxidant enzymes and the contents of non-enzymatic antioxidant substances related to the cellular antioxidant defense system. The hydrogen peroxide (H_2_O_2_) content and superoxide anion (O_2_^-^) generation rate were measured using commercial assay kits (Nanjing Jiancheng Bioengineering Institute, Jiangsu, China), following the manufacturer’s protocols. The activities of glutathione peroxidase (GPX), ascorbate peroxidase (APX) and superoxide dismutase (SOD) were determined via spectrophotometry according to the method described by Wei et al. (2022). The activities of catalase (CAT) and peroxidase (POD) were assayed following the protocol of Cong et al. (2023). The contents of malondialdehyde (MDA), glutathione (GSH) and ascorbate (AsA) were determined referring to Tian et al. (2022). During sampling, the main leaf veins were avoided to ensure sample homogeneity. Each treatment consisted of three independent biological replicates.

### 2.7 RNA sequencing and bioinformatics analysis

On day 14 after maize flowering, three uniformly growing and healthy plants were randomly selected from each experimental plot, and the ear leaves were sampled. These collected leaves were rapidly frozen in liquid nitrogen and stored at -80 °C for transcriptome sequencing. Total RNA was extracted from the ear leaf samples using a TRIzol reagent kit (Invitrogen, Carlsbad, CA, USA). The quality and integrity of the isolated RNA were assessed using an Agilent 2100 Bioanalyzer (Agilent Technologies, Palo Alto, CA, USA) and RNase-free agarose gel electrophoresis, respectively (Ju et al., 2023). The extracted mRNA was enriched using mRNA Capture Beads. After purification with beads, the mRNA was fragmented under high temperature conditions. The fragmented mRNA was then used as a template to synthesize the first strand of cDNA in a reverse transcription enzyme mixture system. Subsequently, sequencing adapters were ligated to the double-stranded cDNA fragments, and Hieff NGS^®^ DNA Selection Beads were used for fragment purification and target fragment selection. PCR library amplification was then performed, and finally, detection was carried out using the Illumina Novaseq X Plus (Gene Denovo Biotechnology Co, Guangzhou, China).

Clean reads were generated by removing raw reads containing adapter sequences, reads with more than 10% ambiguous N bases, and low-quality reads with more than 50 bases exhibiting a quality score of Q ≤ 20. The high-quality clean reads were then mapped to the maize reference genome using HISAT2 2.1.0, followed by transcript assembled with StringTie v1.3.1. The expression level of each gene was quantified by calculating the FPKM (fragment per kilobase of transcript per million mapped reads) value using RSEM software. Differential expression analysis of genes was performed between the two comparison groups using the DESeq2 software. Genes with the parameter of false discovery rate (FDR) ≤ 0.05 and absolute fold change ≥ 2 were identified as differentially expressed genes (DEGs). Functional enrichment and pathway annotation of DEGs were conducted based on the Kyoto Encyclopedia of Genes and Genomes (KEGG) (http://www.genome.jp/kegg/). The original P-values from KEGG enrichment analysis were adjusted via FDR correction, and pathways with FDR ≤ 0.05 were regarded as significantly enriched metabolic or signaling pathways.

### 2.8 Statistical analysis

Data were processed using Microsoft Excel 2021 (Microsoft Corp., Redmongd, WA, USA), and variance analysis was performed using IBM SPSS Statistics 22.0 software (IBM Corp., Ammonk, N.Y., USA). The least significant difference (LSD) test at a significance level of p < 0.05 was computed using analysis by one-way analysis of variance (ANOVA). The graphs were constructed using Origin 2023 software (OriginLab Inc., Northampton, Massachusetts., USA). Data in graphs are presented as means ± standard deviation (SD) of triplicate biological replicates. The gene expression heatmap was visualized using the online drawing tool (https://www.omicshare.com).

## 3 Results

### 3.1 Effect of exogenous melatonin on maize yield traits under high-temperature stress

**Figure 2:**
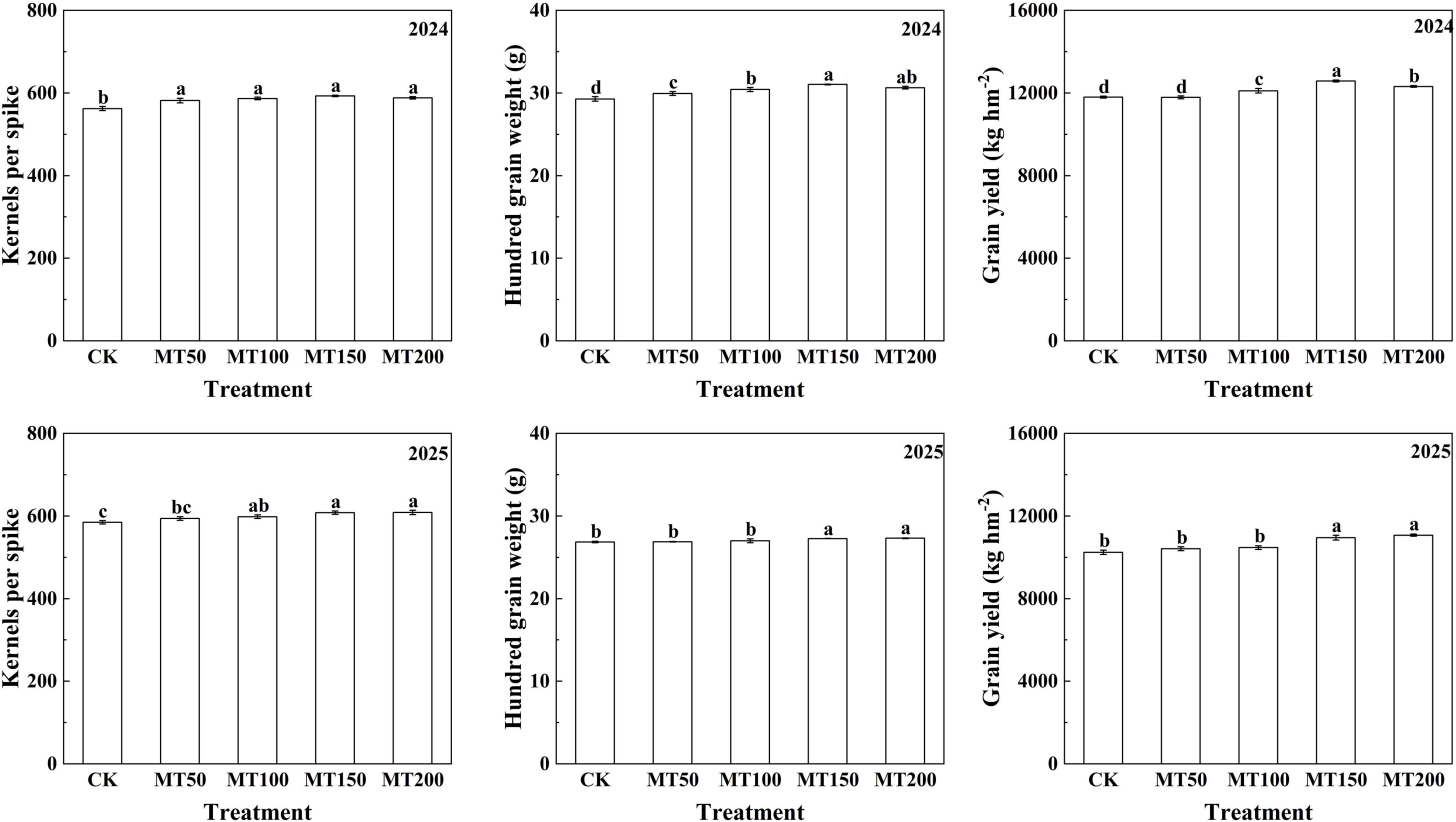
Effect of exogenous melatonin application on yield traits of maize under natural post-flowering high-temperature stress. CK, 0 µmol L^-1^; MT50, 50 µmol L^-1^ melatonin; MT100, 100 µmol L^-1^ melatonin; MT150, 150 µmol L^-1^ melatonin; MT200, 200 µmol L^-1^ melatonin. Data represent means ± SD from triplicate biological replicates. To compare the means, the least significant difference (LSD) test was computed using analysis of variance (ANOVA). Different letters represent significant differences at p < 0.05.

As shown in **Figure 2**, exogenous melatonin treatment significantly increased the number of grains per ear, 100-grain weight and grain yield of maize under high-temperature stress. In 2024, compared with the control treatment (CK), the number of grains per ear under MT50, MT100, MT150 and MT200 treatments was significantly increased by 3.43%, 4.32%, 5.46% and 4.61% respectively, with no significant difference observed among the four melatonin treatments. The 100-grain weight was significantly increased by 2.23%, 3.94%, 6.01% and 4.68% respectively, and no significant difference was detected between MT100, MT150 and MT200 treatments. Compared to CK, maize grain yield did not differ significantly under MT50 treatment, but was significantly increased by 2.62%, 6.62% and 4.38% under MT100, MT150 and MT200 treatments, respectively.

In 2025, the number of grains per ear under MT50, MT100, MT150 and MT200 treatments was significantly higher than that under CK by 1.55%, 2.33%, 3.97% and 4.06%, respectively, with no significant differences among MT100, MT150 and MT200 treatments. The 100-grain weight showed no significantly differences under MT50 and MT100 treatments compared with CK, but was significantly increased by 1.56% and 1.70% under MT150 and MT200 treatments, respectively, with no significant difference between these two treatments. Similarly, no significant difference in grain yield was found between CK, MT50 and MT100 treatments, whereas maize grain yield under MT150 and MT200 treatments was significantly higher than that under CK by 6.88% and 8.06%, respectively, with no significant difference between the two treatments.

### 3.2 Effect of exogenous melatonin on agronomic traits of maize under high-temperature stress

Plant and ear agronomic traits of mature maize were measured in this study. As shown in **Table 1**, exogenous melatonin application modulated maize agronomic traits under high-temperature stress. In 2024, compared with the CK treatment, maize plant height showed no significant differences under MT50 and MT100 treatments, but was significantly increased by 5.13% and 2.03% under MT150 and MT200 treatments, respectively. Ear height did not differ significantly under MT50, MT100, and MT150 treatments relative to CK treatment, but was significantly increased by 12% under MT200 treatment. All four melatonin treatments significantly increased stem diameter by 12.39%, 12.35%, 11.92% and 19.80%, respectively, with no significant differences among treatments. In 2025, plant height was significantly increased by 5.11%, 12.35%, 11.92% and 19.80% under MT50, MT100, MT150 and MT200 treatments, respectively, compared with CK. Ear height was correspondingly elevated by 20.64%, 13.87%, 21.83% and 20.64%, respectively, with no significant differences among the four treatments. Stem diameter showed no significant differences under MT50 and MT100 treatments, but was significantly increased by 5.67% and 5.77% under MT150 and MT200 treatments, respectively.

**Table 1:** Effects of exogenous melatonin application on agronomic traits of maize under post-flowering natural high-temperature stress.

Treatment	Plant height (cm)	Ear height (cm)	Stem diameter (mm)	Ear length (cm)	Ear diameter (mm)	Cob diameter (mm)
2024						
CK	224.77 ± 0.67c	85.27 ± 2.41b	19.29 ± 1.15b	15.79 ± 0.10c	46.30 ± 1.18a	23.82 ± 1.04a
MT50	226.50 ± 1.00c	85.17 ± 1.04b	21.68 ± 1.55a	15.97 ± 0.21bc	46.78 ± 1.77a	23.82 ± 1.32a
MT100	226.20 ± 0.92c	86.17 ± 1.04b	21.67 ± 1.19a	16.13 ± 0.15ab	46.03 ± 1.82a	22.62 ± 1.73a
MT150	236.30 ± 1.01a	90.00 ± 1.32ab	21.59 ± 1.11a	16.28 ± 0.07a	46.47 ± 1.90a	22.92 ± 1.19a
MT200	229.33 ± 1.15b	95.50 ± 0.62a	23.11 ± 0.75a	16.27 ± 0.06a	46.31 ± 1.92a	23.36 ± 1.67a
2025						
CK	238.23 ± 1.27b	98.50 ± 2.08b	20.57 ± 0.08b	16.46 ± 0.06b	48.33 ± 0.49b	26.91 ± 0.49b
MT50	250.40 ± 0.97a	118.83 ± 4.33a	20.80 ± 0.24ab	16.49 ± 0.14b	48.90 ± 0.42ab	27.22 ± 0.42b
MT100	255.10 ± 3.89a	112.17 ± 1.16a	21.43 ± 0.13ab	17.00 ± 0.07a	49.47 ± 0.35a	27.72 ± 0.35ab
MT150	255.67 ± 5.09a	120.00 ± 4.25a	21.73 ± 0.34a	17.15 ± 0.07a	49.89 ± 0.41a	28.40 ± 0.41a
MT200	257.33 ± 1.36a	118.83 ± 2.46a	21.75 ± 0.37a	17.31 ± 0.07a	49.51 ± 0.38a	28.37 ± 0.38a

CK, 0 µmol L^-1^; MT50, 50 µmol L^-1^ melatonin; MT100, 100 µmol L^-1^ melatonin; MT150, 150 µmol L^-1^ melatonin; MT200, 200 µmol L^-1^ melatonin. Data represent means ± SD from triplicate biological replicates. To compare the means, the least significant difference (LSD) test was computed using analysis of variance (ANOVA). Different letters represent significant differences at p < 0.05.

Major ear agronomic traits at maturity were also investigated. As shown in **Table 1**, in 2024, MT50 treatment had no significant effect on ear length compared to CK treatment, while MT100, MT150, and MT200 treatments significantly increased ear length by 2.17%, 3.10%, and 3.02%, respectively, with no significant differences among the three treatments. No significant variations in ear diameter and cob diameter were observed under all melatonin treatments. In 2025, ear length did not differ significantly under MT50 treatment compared with CK, but, was significantly increased by 3.28%, 4.21%, and 5.14% under MT100, MT150 and MT200 treatments, respectively. Ear diameter was significantly increased by 2.38%, 3.24%, and 2.45% under the three treatments, respectively. Cob diameter showed no significant differences under MT50 and MT100 treatments, but was significantly increased by 5.51% and 5.43% under MT150 and MT200 treatments, respectively. No significant differences were detected between the respective comparable treatments.

### 3.3 Effect of exogenous melatonin on biomass accumulation in maize under high-temperature stress

**Figure 3:**
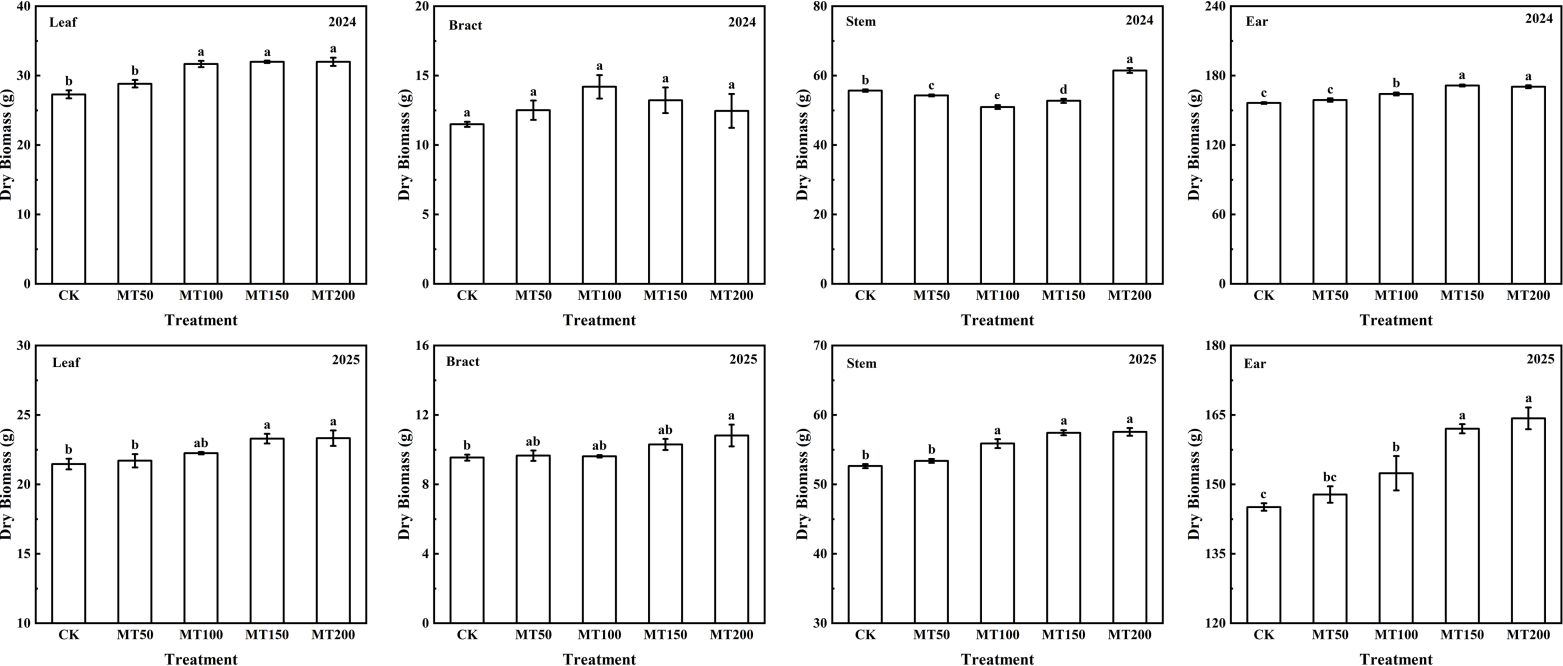
The effect of exogenous melatonin application on dry matter accumulation in different maize organs under post-flowering natural high-temperature stress. CK, 0 µmol L^-1^; MT50, 50 µmol L^-1^ melatonin; MT100, 100 µmol L^-1^ melatonin; MT150, 150 µmol L^-1^ melatonin; MT200, 200 µmol L^-1^ melatonin. Data represent means ± SD from triplicate biological replicates. To compare the means, the least significant difference (LSD) test was computed using analysis of variance (ANOVA). Different letters represent significant differences at p < 0.05.

As shown in **Figure 3**, in 2024, leaf dry matter content of maize under MT50 treatment showed no significant difference compared to the CK treatment, whereas that under MT100, MT150 and MT200 treatments was significantly increased by 16.03%, 17.21% and 17.23%, respectively, with no significant differences among the three treatments. Maize main stem dry matter content was significantly elevated by 10.37% under MT200 treatment compared with CK treatment. No significant differences in bracts dry matter content were observed across all melatonin treatments relative to CK. Ear dry matter content showed no significant change under MT50 treatment, but was significantly increased by 4.96%, 9.63% and 9.00% under MT100, MT150 and MT200 treatments, respectively, with no significant difference between MT150 and MT200 treatments. In 2025, leaf dry matter content did not differ significantly under MT50 and MT100 treatments compared with CK, but was significantly increased by 8.54% and 8.70% under MT150 and MT200 treatments, respectively, with no significant difference between the two treatments. Furthermore, main stem dry matter content showed no significantly variation under MT50 treatment, yet was significantly elevated by 6.16%, 9.13%, and 9.38% under MT100, MT150 and MT200 treatments, respectively, with no significant differences among the three treatments. Additionally, bract dry matter weight showed no significant differences under the MT50, MT100 and MT150 treatments relative to CK, but was significantly increased by 13.27% under MT200 treatment. Ear dry matter content did not differ significantly under MT50 treatment, but significantly enhanced by 5.02%, 11.67%, and 13.21% under MT100, MT150 and MT200 treatments, respectively, with no significant difference between MT150 and MT200 treatments.

### 3.4 Effects of exogenous melatonin on photosynthetic characteristics of maize ear leaves under high-temperature stress

**Figure 4:**
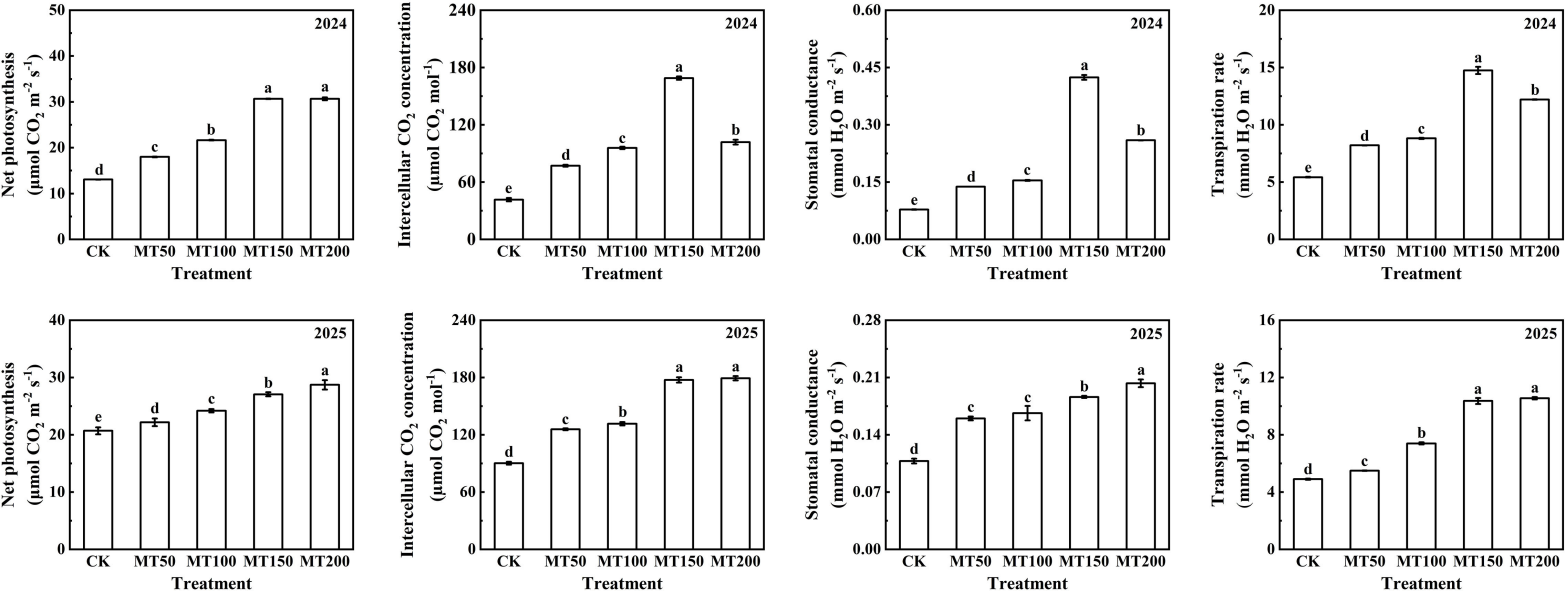
Effect of exogenous melatonin application on photosynthetic characteristics of maize ear leaves under post-flowering natural high temperature stress. CK, 0 µmol L^-1^; MT50, 50 µmol L^-1^ melatonin; MT100, 100 µmol L^-1^ melatonin; MT150, 150 µmol L^-1^ melatonin; MT200, 200 µmol L^-1^ melatonin. Data represent means ± SD from triplicate biological replicates. To compare the means, the least significant difference (LSD) test was computed using analysis of variance (ANOVA). Different letters represent significant differences at p < 0.05.

In 2024, compared with CK treatment, the net photosynthetic rate of maize ear leaves under the MT50, MT100, MT150 and MT200 treatments was significantly increased by 37.66%, 65.74%, 134.66% and 134.63%, respectively, no significant difference was observed between MT150 and MT200 (**Figure 4**). Intercellular CO_2_ concentration increased significantly by 85.88%, 130.75%, 306.89% and 145.37%, respectively. while stomatal conductance increased significantly by 72.62%, 95.43%, 441.19% and 230.11%, and transpiration rate increased by 51.37%, 62.63%, 172.16% and 125.20%, correspondingly. In 2025, the net photosynthetic rate under the four melatonin treatments was significantly higher than CK by 7.19%, 17.01%, 30.88% and 38.84%, respectively. Intercellular CO_2_ concentration significantly increased by 39.45%, 45.83%, 96.68% and 98.74%, respectively, with no significant difference between MT150 and MT200. Furthermore, the stomatal conductance was significantly increased by 48.15%, 54.63%, 72.22% and 87.96% and transpiration rate increased by 12.04%, 51.08%, 111.43% and 115.37%, respectively, but there was no significant difference between MT150 and MT200 treatments. Overall, the two-years trends in leaf gas exchange parameters were consistent.

### 3.5 Effects of exogenous melatonin on key antioxidant substances contents in maize ear leaves under high-temperature stress

**Figure 5:**
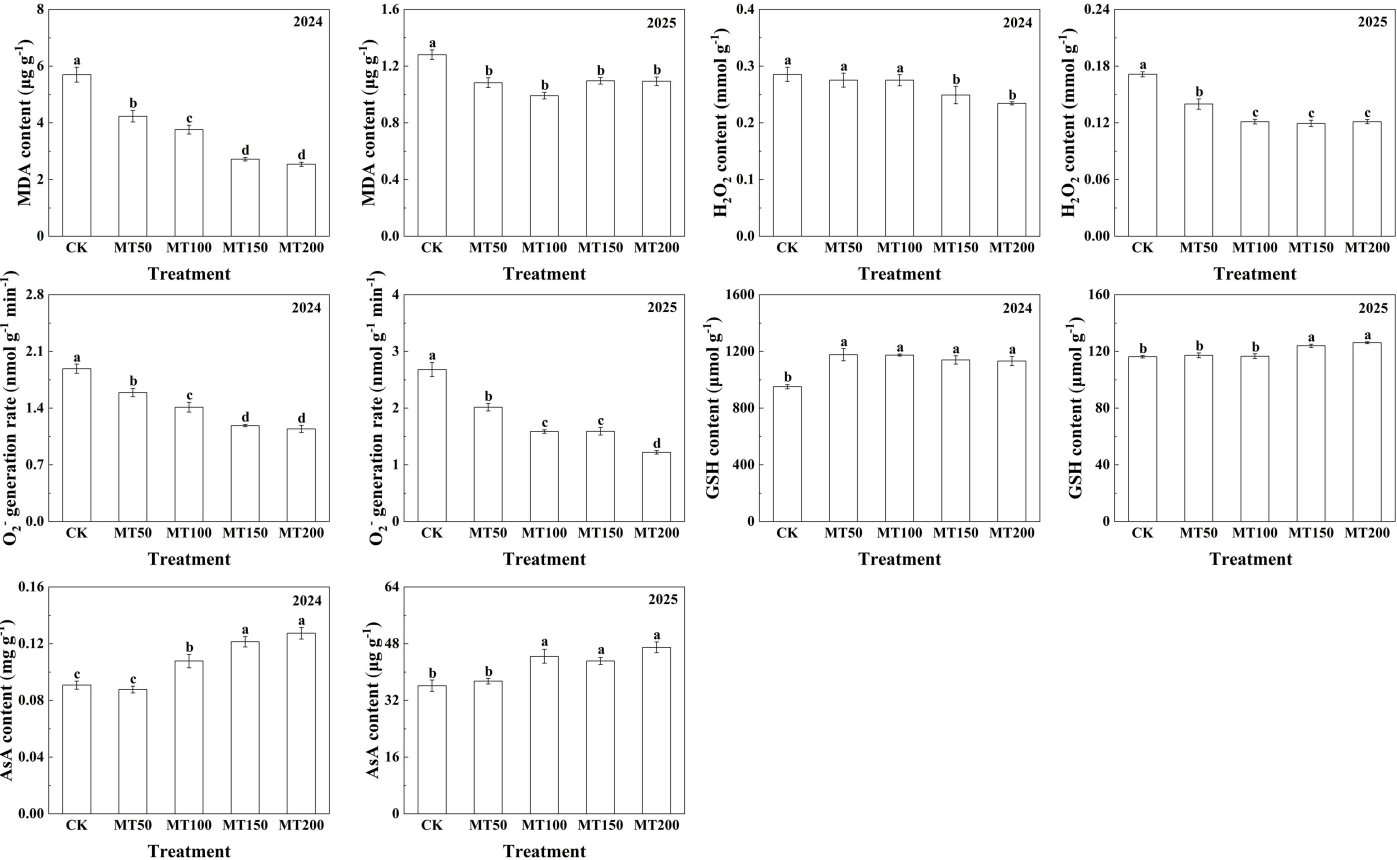
Effects of exogenous melatonin application on key antioxidant substances contents in maize ear leaves under post-flowering natural high-temperature stress. CK, 0 µmol L^-1^; MT50, 50 µmol L^-1^ melatonin; MT100, 100 µmol L^-1^ melatonin; MT150, 150 µmol L^-1^ melatonin; MT200, 200 µmol L^-1^ melatonin. Data represent means ± SD from triplicate biological replicates. To compare the means, the least significant difference (LSD) test was computed using analysis of variance (ANOVA). Different letters represent significant differences at p < 0.05.

To elucidate the regulatory effects of exogenous melatonin at various concentrations on the leaf antioxidant system of maize under high-temperature stress, key antioxidant substances contents in maize ear were determined in this study (**Figure 5**). Specifically, in 2024, compared with CK, malondialdehyde (MDA) contents in maize ear leaves were significantly reduced by 25.74%, 34.08%, 52.36%, and 55.45% under the treatments of MT50, MT100, MT150, and MT200, respectively, with no significant difference between MT150 and MT200 treatments. Hydrogen peroxide (H_2_O_2_) contents showed no significant variations under MT50 and MT100 treatments relative to CK, but were significantly reduced by 12.59% and 17.82% under MT150 and MT200 treatments, respectively, with no significant difference between the two treatments. Meanwhile, superoxide anion (O_2_^-^) generation rate was significantly decreased by 15.61%, 25.21%, 37.18%, and 39.42% under the four melatonin treatments, respectively, with no significant difference between MT150 and MT200 treatments. In 2025, malondialdehyde contents were significantly decreased by 15.44%, 22.53%, 14.37% and 14.63% under MT50, MT100, MT150 and MT200 treatments, respectively, compared with CK. Hydrogen peroxide contents were significantly decreased by 18.40%, 29.24%, 30.32% and 29.22%, respectively, with no significant differences among MT100, MT150, and MT200 treatments. Superoxide anion production rate was notably declined by 24.89%, 40.78%, 40.64% and 54.52% under the four melatonin treatments, respectively.

In 2024, glutathione (GSH) contents in maize ear leaves were significantly increased by 23.78%, 23.52%, 19.86%, and 19.03% under MT50, MT100, MT150, and MT200 treatments, respectively, with no significant differences among the four treatments. Ascorbic acid (AsA) content showed no significantly difference under MT50 treatment compared with CK, but was significantly elevated by 18.40%, 33.32%, and 39.95% under MT100, MT150 and MT200 treatments, respectively, and with no significant difference between MT150, MT200 treatments. In 2025, glutathione (GSH) contents exhibited no significant changes under MT50 and MT100 treatments compared to CK, but were significantly increased by 6.68%, 8.54% under MT150 and MT200 treatments, respectively, with no significant difference between the two treatments. Similarly, ascorbic acid (AsA) content showed no significant variation under MT50 treatment compared with CK, but was significantly increased by 22.94%, 19.26%, and 29.96% under MT100, MT150 and MT200 treatments, respectively, with no significant differences among the three treatments.

### 3.6 Effects of exogenous melatonin on the activities of key antioxidant enzymes in maize ear leaves under high-temperature stress

**Figure 6:**
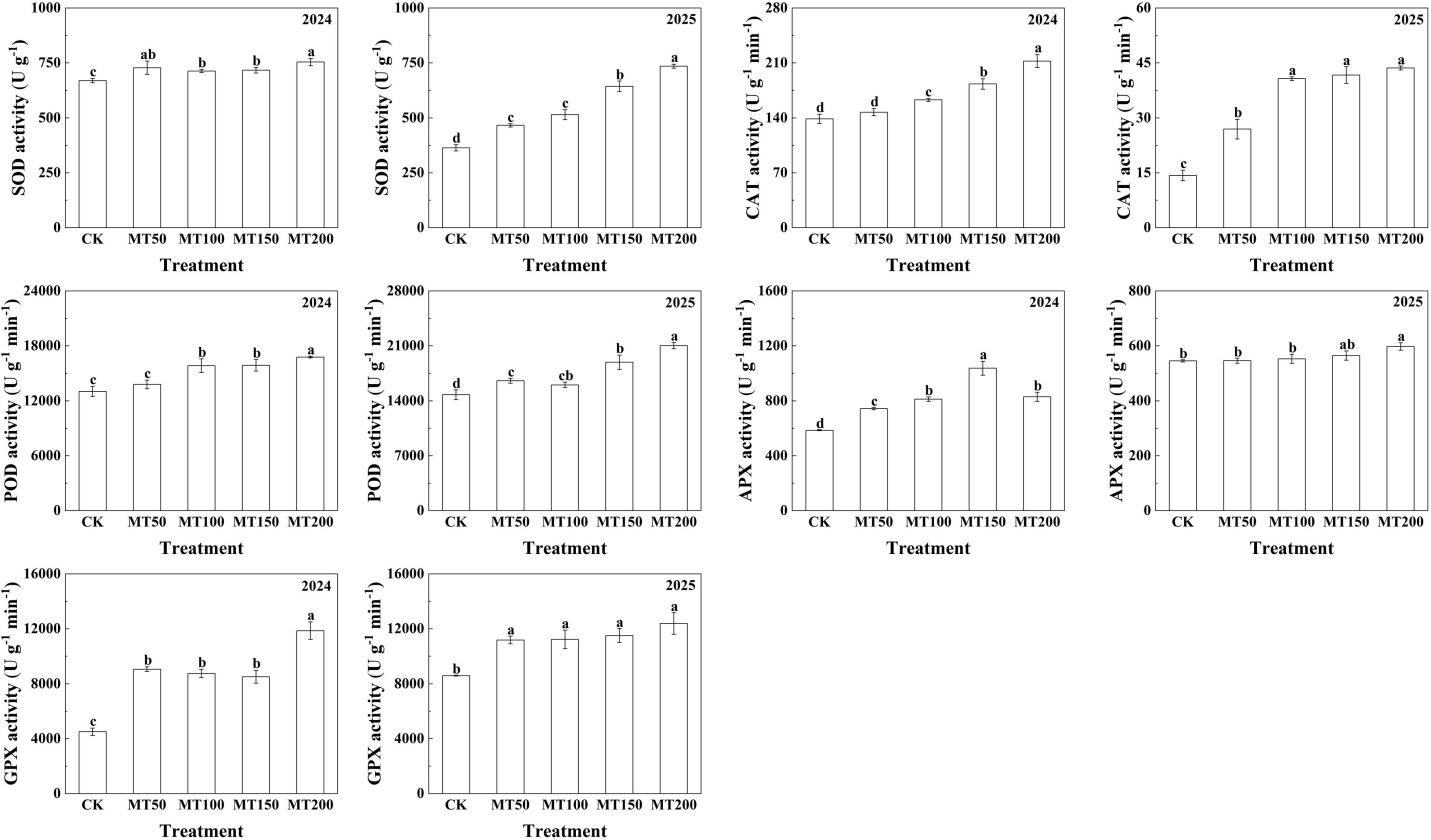
Effects of exogenous melatonin application on the activities of key antioxidant enzymes in maize ear leaves under post-flowering natural high-temperature stress. CK, 0 µmol L^-1^; MT50, 50 µmol L^-1^ melatonin; MT100, 100 µmol L^-1^ melatonin; MT150, 150 µmol L^-1^ melatonin; MT200, 200 µmol L^-1^ melatonin. Data represent means ± SD from triplicate biological replicates. To compare the means, the least significant difference (LSD) test was computed using analysis of variance (ANOVA). Different letters represent significant differences at p < 0.05.

The activities of key antioxidant enzymes in maize ear leaves were determined across different concentrations (**Figure 6**). Specifically, in 2024, superoxide enzymes (SOD) activities under MT50, MT100, MT150, and MT200 were significantly increased by 5.00%, 6.54%, 7.07%, and 12.63%, respectively, compared to CK. Peroxidase (POD) activity did not differ significantly between MT50 and CK treatment, but increased significantly by 21.50%, 21.95%, and 28.74% under MT100, MT150 and MT200, respectively; catalase (CAT) activities in the four melatonin treatments were significantly increased by 100.96%, 94.15%, 88.76%, and 163.26%, respectively, showing consistent variation with POD. In 2025, ascorbate peroxidase (APX) activities under MT50, MT100, MT150 and MT200 increased significantly by 26.97%, 38.59%, 77.09%, and 41.63%, respectively. Peroxidase activity under MT50, MT150, and MT200 treatments was significantly increased by 11.91%, 27.90%, and 42.27% respectively compared with CK. Catalase was significantly enhanced by 89.10%, 185.68%, 192.51%, and 206.18% across the four treatments, with no significant difference among MT100, MT150 and MT200 treatments. Compared with the CK treatment, the glutathione peroxidase activity was significantly increased by 30.26%, 23.48%, 34.00%, and 44.25%, respectively. Additionally, there was no significant difference in ascorbate peroxidase activity between MT50, MT100 and CK treatments, but increased by 3.60% and 9.64% under MT150 and MT200, with no significant difference between the two treatments.

### 3.7 Effects of exogenous melatonin on the expression of key photosynthetic genes in maize ear leaves under high-temperature stress

**Figure 7:**
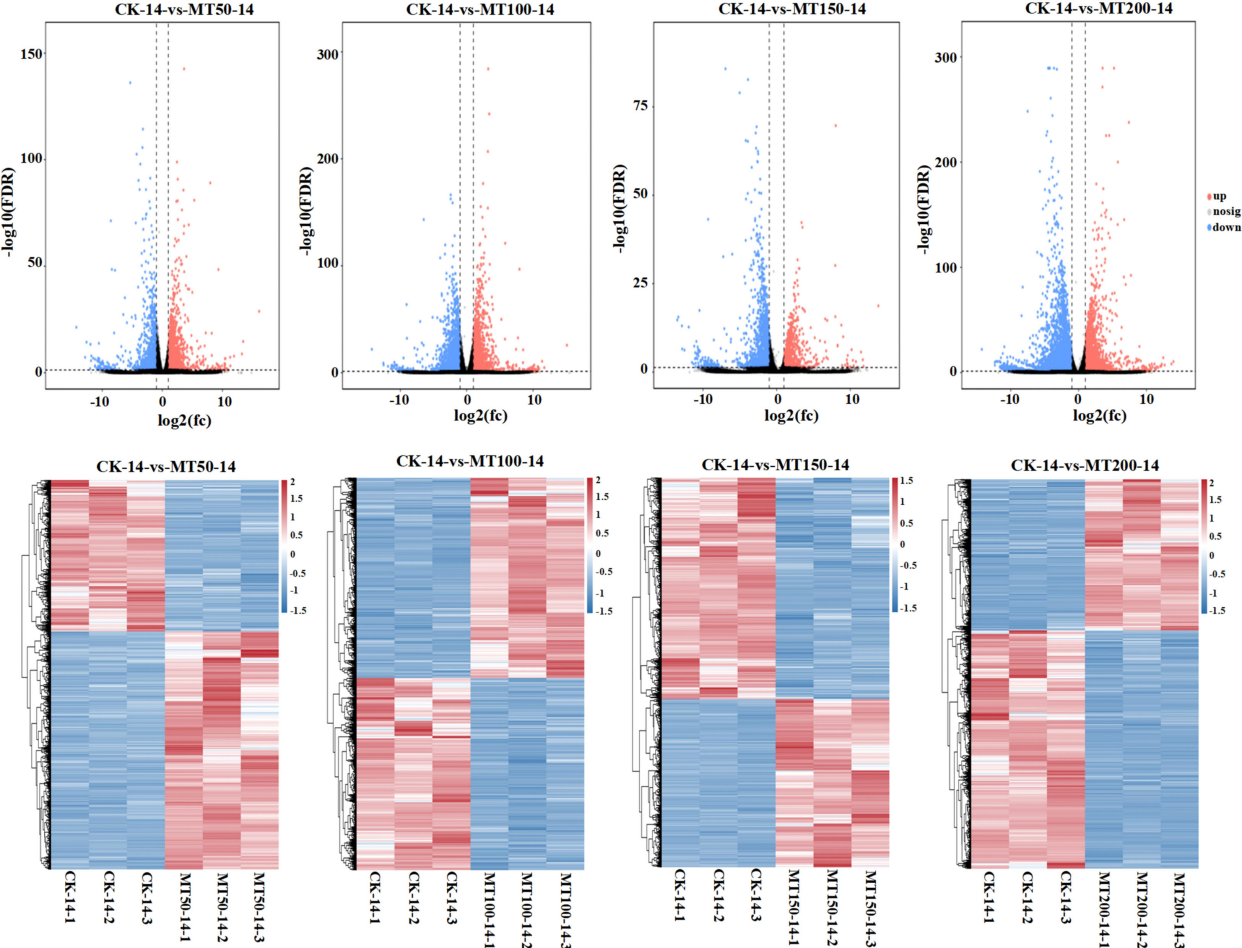
Volcano plot of differentially expressed gene numbers and heatmap visualizing expression trends among different treatments. The picture above shows a volcano plot illustrating the number of differentially expressed genes (DEGs) in comparisons between different melatonin treatments and the control (CK), while the bottom picture displays a heatmap visualizing the expression trends of these DEGs. CK, 0 µmol L^-1^; MT50, 50 µmol L^-1^ melatonin; MT100, 100 µmol L^-1^ melatonin; MT150, 150 µmol L^-1^ melatonin; MT200, 200 µmol L^-1^ melatonin. The color in the heatmap indicates the gene expression. Red represents high expression, and blue represents low expression.

**Figure 8:**
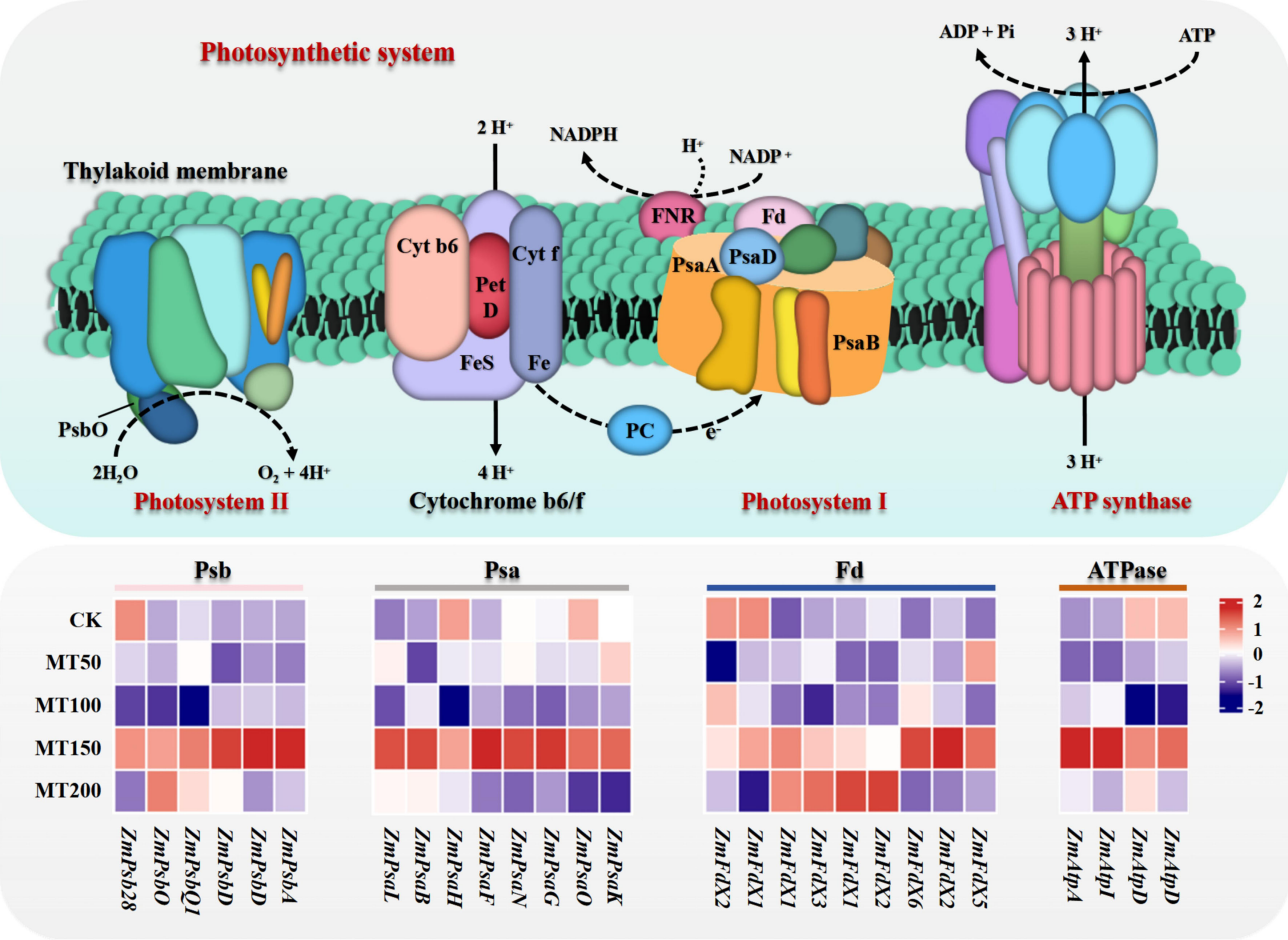
Effects of exogenous melatonin application on key photosynthetic gene expression in maize ear leaves under post-flowering natural high-temperature stress. The picture above shows a schematic diagram of the photosynthetic system, and the bottom picture shows the expression of key genes in the photosynthetic system of maize ear leaves under different concentrations of melatonin treatment in natural high-temperature conditions. CK, 0 µmol L^-1^; MT50, 50 µmol L^-1^ melatonin; MT100, 100 µmol L^-1^ melatonin; MT150, 150 µmol L^-1^ melatonin; MT200, 200 µmol L^-1^ melatonin. ATPase, ATP synthase; Fd, ferredoxin; Psa, chloroplast photosystem I reaction center subunit; Psb, photosystem II reaction center protein. The color in the heatmap indicates the gene expression. Red represents high expression, and blue represents low expression. More detailed information is shown in **Supplementary Table 2**.

To further investigate the regulatory effects of different concentrations of exogenous melatonin on the expression of key genes involved in vital biological pathway in maize, transcriptome sequencing was performed on maize ear leaves at 14 days after flowering under various melatonin treatments. As shown in **Figure 7**, the volcano plot illustrates the number of differentially expressed genes (DEGs), while the heatmap reveals the expression patterns of these genes under different melatonin concentrations relative to the control (CK). The KEGG pathway enrichment analysis of the transcriptome data revealed that the photosynthetic system pathway was significantly enriched in multiple comparison groups. In accordance with the screening standard of FPKM ≥ 10 for gene expression abundance across all samples, 27 differentially expressed genes (DEGs) significantly enriched in the photosynthetic pathway were identified. Among these DEGs, 4 DEGs were annotated as ATP synthase (ATPase), 9 as ferredoxin (Fd), 8 as chloroplast photosystem I reaction center subunit (Psa), and 6 as photosystem II reaction center proteins (Psb). The heatmap visualization analysis of the expression levels of DEGs (**Figure 8**) demonstrated that most of the genes encoding ATPase, Psa, Psb, and Fd in photosynthesis system were highly expressed under MT150 treatment compared with CK. In particular, the transcript levels of genes associated with photosystem II reaction center were significantly upregulated under MT150 treatment compared with CK. Taken together, foliar application of 150 µmol L^-1^ melatonin significantly enhanced the transcription of key photosynthetic genes in maize ear leaves, which contributed to the improvement of photosynthesis capacity in maize plants, thereby alleviating the physiological damage induced by post-flowering high temperature stress.

### 3.8 Effects of exogenous melatonin on the expression of key genes in the reactive oxygen species scavenging system of maize ear leaves under high-temperature stress

**Figure 9:**
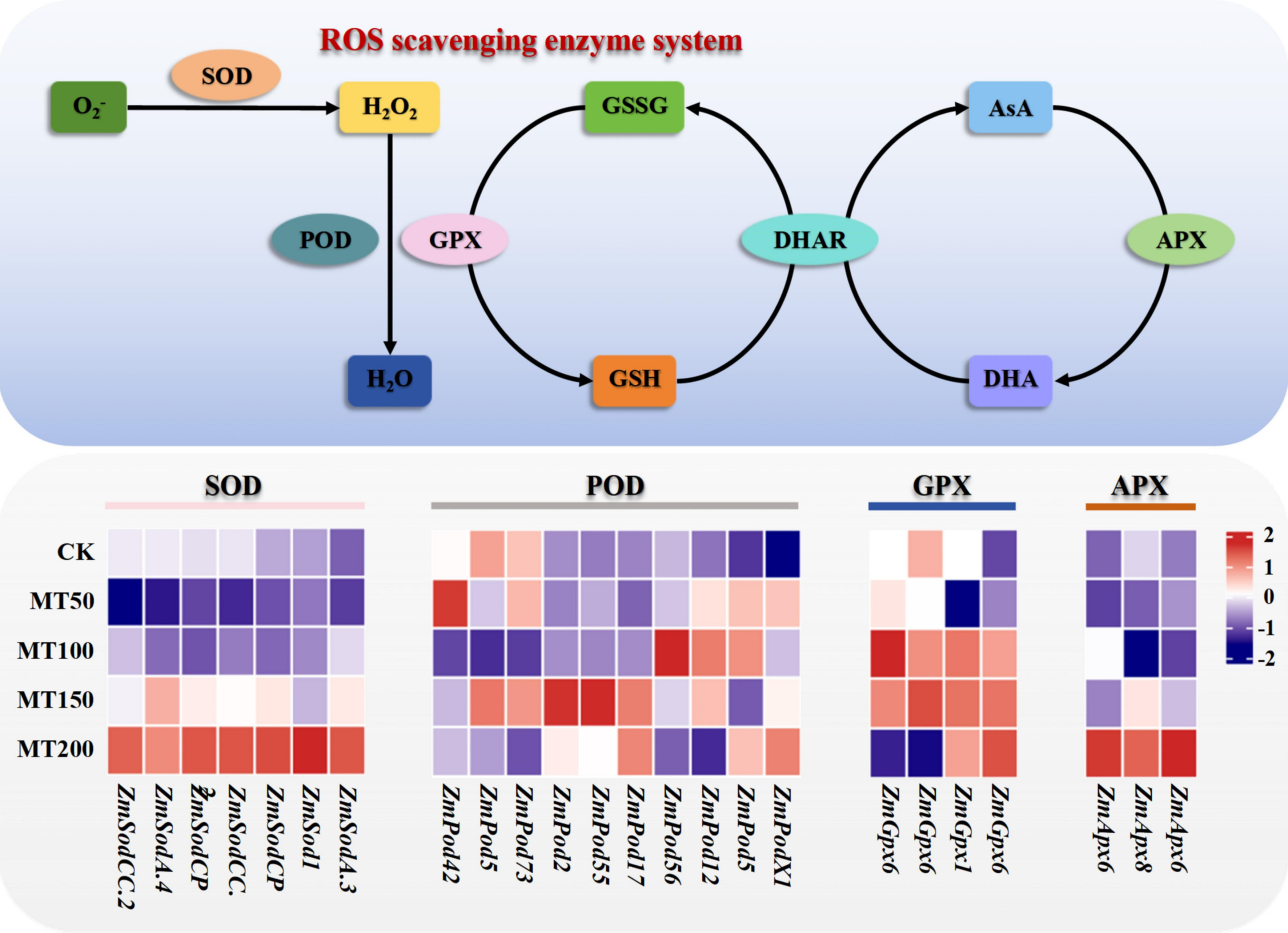
Effects of exogenous melatonin application on the expression of key genes associated with the ROS scavenging system in maize ear leaves under post-flowering natural high-temperature stress. The picture above shows a schematic diagram of the reactive oxygen species scavenging system, and the bottom picture shows the expression of key genes in the reactive oxygen species scavenging system of maize ear leaves under different concentrations of melatonin treatment in natural high-temperature conditions. CK, 0 µmol L^-1^; MT50, 50 µmol L^-1^ melatonin; MT100, 100 µmol L^-1^ melatonin; MT150, 150 µmol L^-1^ melatonin; MT200, 200 µmol L^-1^ melatonin. SOD, superoxide dismutase; POD, Peroxidase; APX, ascorbate peroxidase; GPX, glutathione peroxidase. The color in the heatmap indicates the gene expression. Red represents high expression, and blue represents low expression. More detailed information is shown in **Supplementary Table 3**.

In view of the significant variations in reactive oxygen species (ROS) generation and scavenging-related substance contents and enzyme activities in maize ear leaves following treatment with different concentrations of melatonin, the expression levels of key enzyme genes involved in the ROS scavenging system and ascorbic acid-glutathione cycle were further analyzed in this study. After filtering and screening, a total of 24 key genes were identified. Among these genes, 10 were annotated as peroxidases (POD), 7 as superoxide dismutases (SOD), 3 as ascorbic acid peroxidases (APX), and 4 as glutathione peroxidases (GPX). As shown in **Figure 9**, overall, melatonin treatment overall upregulated the expression levels of key genes encoding enzymes in the ROS scavenging system. Specifically, the transcript levels of most key enzyme genes related to the ROS scavenging pathway were significantly higher in maize ear leaves under MT150 and MT200 treatments than those under the CK treatment. The expression levels of POD and GPX encoding genes reached the peak under MT150 treatment. These results suggest that exogenous melatonin at an appropriate concentration can elevate the expression of key enzyme genes related to the ROS scavenging system in maize leaves, thereby enhancing the corresponding antioxidant enzyme activities and improving the resistance of maize to post-flowering high-temperature stress.

## 4 Discussion

### 4.1 Regulatory effects of exogenous melatonin application on maize yield and dry matter accumulation under high-temperature stress

Climate warming has induced frequent, prolonged and intense heat waves, which severely restrict maize growth, development and yield formation (Li et al., 2018). Although maize is a thermophilic crop, excessively high temperatures readily trigger defective pollination and kernel abortion, drastically reducing seed setting rate and causing a 12.4% to 30% yield loss (McNellie et al., 2018). Maize exhibits greater sensitivity to high temperature during reproductive growth than the vegetative growth stage. The optimal daily average temperature for grain filling is 22-24 °C (Kamali et al., 2018). Research by Shao et al. (2021) documented that temperatures exceeding 28 °C suppress normal anther dehiscence in maize, while anthers lose viability and desiccate at 32-35 °C; persistent high temperature above 35 °C disrupts anther development and shortens the pollen dispersal period.

In recent years, global climate warming has triggered frequent extreme high-temperature events from July to August across the Huang-Huai-Hai Plain, featuring high intensity, long duration and strong instability (Tian et al., 2018; Wang et al., 2018). Statistics reveal that high-temperature stress occurred in seven years during 2013 to 2022 in this region, especially in 2022, some regions experienced temperatures above 35 °C for more than 35 days. However, with the addition of an extreme heat day, maize production decreases by 226.62 kg hm^-2^ (Zhang and Yang, 2019). The continuous high temperature and drought weather have severely restricted the increase of maize yield. Thus, persistent high temperature has become a major limiting factor for maize yield improvement, and developing feasible cultivation strategies to alleviate heat-induced damage is critical for securing high and stable maize production in the Huang-Huai-Hai Plain. Accumulating evidence confirms that melatonin effectively promotes plant growth and maintains the homeostasis of stress defense systems, playing a vital regulatory role in plant abiotic stress tolerance (Posmyk et al., 2009; Xia et al., 2020).

In this two-year field experiment, we systematically evaluated the regulatory effects of different concentrations of exogenous melatonin on yield traits and organ-level dry matter accumulation in mature maize. Results showed that exogenous melatonin application significantly increased grain number per ear and 100-grain weight, thereby substantially boosting maize yield. In 2024, maize yield under MT150 and MT200 treatments was 6.62% and 4.38% higher than that of CK, respectively; in 2025, the two treatments elevated yield by 6.88% and 8.06%, respectively (**Figure 2**). Studies on other crops have also verified that high-temperature stress impedes biomass accumulation and yield formation, whereas optimal concentrations of exogenous melatonin effectively mitigate heat damage, facilitate nutrient uptake and assimilate accumulation, and ultimately improve crop yield (Hu et al., 2020; Jahan et al., 2021b; Khattak et al., 2022; Brengi et al., 2025).

The accumulation and distribution of dry matter are of crucial importance for maize formation yield, and total dry matter accumulation is generally positively correlated with yield (Yang et al., 2020). Here, exogenous melatonin application modified dry matter partitioning among maize organs, with pronounced effects detected in the main stem and ears. Compared with CK, MT150 treatment significantly increased ear dry matter accumulation by 9.63% and 11.67% in 2024 and 2025, respectively, with no significant difference between MT150 and MT200 treatments (**Figure 3**), which is consistent with previous studies. Collectively, exogenous melatonin application at 150 µmol L^-1^ and 200 µmol L^-1^ improves grain number and grain weight, promotes assimilate translocation and deposition into kernels, enhances biomass accumulation in maize ears, and ultimately increases grain yield under high-temperature stress.

### 4.2 Regulatory effects of exogenous melatonin application on maize agronomic traits under high-temperature stress

Plant growth is a continuous process involving biomass accumulation and morphological expansion. However, high-temperature stress triggers a series of abnormal physiological and biochemical responses in plants, ultimately restricting plant growth and even inducing plant death. The morphological damage caused by high-temperature stress mainly manifests as reduced photosynthetic leaf area, inhibited biomass accumulation, dwarfed plant height, morphology and accelerated premature senescence (Lipiec et al., 2013). Previous studies have demonstrated that high-temperature stress significantly suppresses plant growth, while melatonin effectively mitigates growth inhibition induced by temperature stress and enhances plant stress tolerance (Hawkins et al., 2013; Hu et al., 2016; Li et al., 2019; Xing et al., 2021; Li et al., 2022a, Li et al., 2022c; Brengi et al., 2025). In this study, the regulatory effects of exogenous melatonin on maize agronomic traits under-high-temperature stress were investigated. The results showed that MT150 and MT200 treatments significantly improved maize plant height, ear height, and stem diameter compared with the CK treatment, with the most prominent enhancement observed in stem diameter (**Table 1**), which is consistent with previous findings. Excessively temperatures during maize growth and development often induce excessive plant elongation and weaken stem lodging resistanceh, thereby exerting adverse impacts on summer maize yield. Here, melatonin significantly alleviated the detrimental effects of high-temperature stress on maize growth. Exogenous melatonin may strengthen the mechanical strength of maize stems by increasing stem diameter, which contributes to constructing a robust plant architecture and improving adaptability to stressful environments. High temperatures accelerate various physiological metabolism and disrupt reproductive development in maize, which is detrimental to pollen formation, resulting in delayed flowering, inhibited pollen dispersal, reduced pollen viability, defective pollination, decreased fertile kernel number and shriveled kernels. Additionally, prolonged high temperature shortens ear length, reduces ear diameter and cob diameter, lowers grain number per ear and decreases grain weight, thereby severely restricting maize yield. This study revealed that MT150 and MT200 treatments significantly increased maize ear length, ear diameter and cob diameter relative to CK (**Table 1**). Combined with yield component data, melatonin treatment effectively optimized ear morphology traits, and significantly increased grain number per ear and 100-grain weight. These results indicate that melatonin enhances the tolerance of maize to post-flowering high-temperature stress, promotes healthy ear development, improves plant growth and grain filling, and ultimately increases maize yield under high-temperature stress.

### 4.3 Regulatory effects of exogenous melatonin application on the photosynthetic system of maize leaves under high-temperature stress

High temperature directly disrupts electron transfer and thylakoid membrane structure in plants, thereby impairing the stability of the photosynthetic system and causing irreversible physiological damage (Rotundo et al., 2019; Alemu, 2020). Long-term exposure to high temperature triggers dysfunction of the maize photosynthetic system, characterized by reduced stomatal conductance, decreased transpiration rate and elevated intercellular CO_2_ concentration. This further inhibits photosynthetic electron transport and weakens photosynthetic performance, severely restricting grain filling and yield formation in maize (Mathur et al., 2018; Zhang et al., 2022). Previous studies have demonstrated that high-temperature stress suppresses photosynthesis by reducing the oxidation activity of PSII receptors and lowering electron transport efficiency in both PSI and PSII. In addition, chloroplast structural and functional damage is also a critical factor limiting the photosynthetic rate of maize under heat stress (Mathur et al., 2014, Mathur et al., 2018; Rotundo et al., 2019; Alemu, 2020; Zhang et al., 2022). Accumulating evidence has verified that melatonin enhances heat tolerance in plants by activating stress-related protein and enzyme pathways, and protecting cellular physiological activity (Shi et al., 2015; Qi et al., 2018). For instance, Jahan et al. (2021a) reported that melatonin promoted CO_2_ assimilation, enhanced Rubisco and FBPase activities, and upregulated the transcription of photosynthesis-related genes in tomato seedlings under high-temperature stress.

In the present study, MT150 and MT200 treatments significantly elevated the net photosynthetic rate, stomatal conductance, transpiration rate and intercellular CO_2_ concentration of maize ear leaves, suggesting that optimal concentrations of melatonin effectively improve photosynthetic performance, alleviate the inhibition of heat stress on photosynthesis, and mitigate high-temperature-induced damage in maize. Furthermore, transcriptomic analysis revealed that photosynthesis-related pathways were significantly enriched in the melatonin vs. CK comparison groups. Notably, MT150 treatment upregulated the expression of most genes encoding ATPase, Psa, Psb and Fd in the photosynthetic system, especially the genes related to PSI and PSII reaction centers. These results indicate that 150 µmol L^-1^ melatonin promotes the transcription of key photosynthetic genes in maize ear leaves, thereby enhancing plant photosynthetic capacity and alleviating post-flowering high-temperature damage. This finding is consistent with a previous study that exogenous melatonin improves heat tolerance in tomato seedlings by maintaining chlorophyll metabolism and photosynthetic stability (An et al., 2025).

### 4.4 Regulatory effects of exogenous melatonin application on the antioxidant defense system of maize leaves under high-temperature stress

High temperature severely damages plant cellular structure and induces lipid peroxidation of cell membranes, resulting in reduced antioxidant enzyme activity and excessive accumulation of ROS. Excessive ROS accumulation inhibits protein synthesis and accelerates the production of O_2_^-^ and H_2_O_2_ in plant cells (Zhao et al., 2016). Additionally, elevated ROS levels oxidize saturated fatty acids in plant thylakoid membranes, leading to a marked increase in MDA content, impairing the structural and functional integrity of biological membranes, and ultimately causing irreversible cellular damage (Anwar et al., 2018). Melatonin has been widely verified to play a vital role in enhancing plant antioxidant capacity, maintaining cell membrane stability, restraining chlorophyll degradation, regulating stomatal function and alleviating abiotic stress damage (Arnao and Josefa, 2007; Ding et al., 2018; Wei et al., 2018; Wang et al., 2020; Zhang et al., 2021a). A wealth of studies has demonstrated that melatonin enhances stress resistance in various plants by promoting the accumulation of osmoregulatory substances, reducing ROS overproduction, and improving the activity of antioxidant enzymes (Weeda et al., 2014; Manchester et al., 2015; Dai et al., 2020; Liu et al., 2020; Zhang et al., 2021b). Kolupaev et al. (2024) reported that melatonin enhances heat tolerance in wheat seedlings by activating the ROS scavenging system. Buttar et al. (2020) found that melatonin activates the antioxidant system by elevating superoxide dismutase (SOD) activity, decreasing MDA and H_2_O_2_ contents and O_2_^-^ production rate, thus maintaining normal plant growth under high-temperature stress.

Similarly, Li et al. (2019) documented that foliar application of melatonin significantly improved the survival rate of maize seedlings under heat stress, enhanced the activities of CAT and glutathione reductase (GR), reduced MDA accumulation, and alleviated high-temperature-induced damage. In line with these previous findings, the present study revealed that melatonin treatment significantly increased the contents of GSH and AsA in maize leaves, and boosted the activities of key antioxidant enzymes including SOD, CAT, POD, GPX and APX. Transcriptomic analysis further confirmed that MT150 treatment upregulated the expression of key genes encoding antioxidant enzymes in maize ear leaves. Collectively, exogenous melatonin activates the antioxidant defense system in maize by promoting the transcription of key enzyme genes related to ROS scavenging, reduces excessive ROS accumulation, and enhances cellular antioxidant capacity, thereby mitigating oxidative damage triggered by high-temperature stress. Finally, the responsive mechanisms of melatonin in enhancing heat tolerance across different plant species in recent years were summarized in **Table 2**, which provides a theoretical basis for exploring the regulatory effects of melatonin on heat resistance mechanisms in various crops, and offers technical references for stress-resistant cultivation of crops.

**Table 2:** Summary of recent studies on melatonin-mediated regulation of plant responses to post-flowering natural high-temperature stress.

Plant species	Regulated mechanism	Reference
Tomato (*Solanum lycopersicum*)	Enhance the transcription and activities of antioxidant enzymes	Qi et al., 2018
Kiwifruit (*Actinidia deliciosa*)	Promote antioxidant enzymatic activity and glutathione S-transferase transcription	Liang et al., 2018
Maize (*Zea mays* L.)	Modulate antioxidant defense, methylglyoxal detoxification, and osmoregulation systems	Li et al., 2019
Tea (*Camellia sinensis* L.)	Increase epigallocatechin-3-gallate and theanine biosynthesis	Li et al., 2020a
Soybean (*Glycine max* L.)	Balance redox homeostasis and modulating antioxidant defense, phytohormones and polyamines biosynthesis	Imran et al., 2021
Tomato (*Solanum lycopersicum*)	Enhanced the activity of Rubisco and FBPase enzymes, improved the efficiency of electron transfer, and regulated the photosynthetic performance	Jahan et al., 2021a
Chrysanthemum	Reduce the accumulation of reactive oxygen species, enhance antioxidant capacity, and improve photosynthetic efficiency	Xing et al., 2021
Celery (*Apium Graveolens* L.)	Enhance the ability of plant to remove reactive oxygen species in response to heat stress, improve the leaf transpiration and the light energy utilization efficiency	Li et al., 2022b
Tall fescue	Regulate photoelectric conversion of PSII, prevent the production of excessive excitation energy, increased survival rate after heat shock	Wang et al., 2022
Carnation (*Dianthus caryophyllus* L.)	Regulate growth, photosynthetic efficiency and leaf ultrastructure	Hu et al., 2023
Maize (*Zea mays* L.)	Enhance the activity of antioxidant enzymes, increase the content of osmotic regulatory substances, and alleviate the damage to the cell membrane caused by heat stress	Cao et al., 2024
Wheat (*Triticum aestivum* L.)	Enhance the activities of catalase and guaiacol peroxidase, reduce the index of reactive oxygen species generation and the content of lipid peroxidation products, alleviate the cell membrane damage	Kolupaev et al., 2024
Tomato (*Solanum lycopersicum*)	Promote the accumulation of osmotic regulatory substances and enhance the osmotic regulatory capacity	Mumithrakamatchi et al., 2024
Chinese cabbage	Enhance photosynthetic activity, improve the capacity to mitigate oxidative stress	Teng et al., 2024
Tomato (*Solanum lycopersicum*)	Enhance the activity of antioxidant enzymes, protect the chlorophyll metabolism, and improve the photosynthetic capacity	An et al., 2025
Maize (*Zea mays* L.)	Enhance antioxidant defense capacity, promote osmotic regulation, increase proteolytic enzyme activity, and maintain hormone homeostasis	Chen et al., 2025
Buckwheat	Promote the growth, coordinate and regulate the antioxidant enzyme system and osmotic regulatory substances, improve the gas exchange parameters of photosynthesis	Tian et al., 2025

## 5 Conclusions

**Figure 10:**
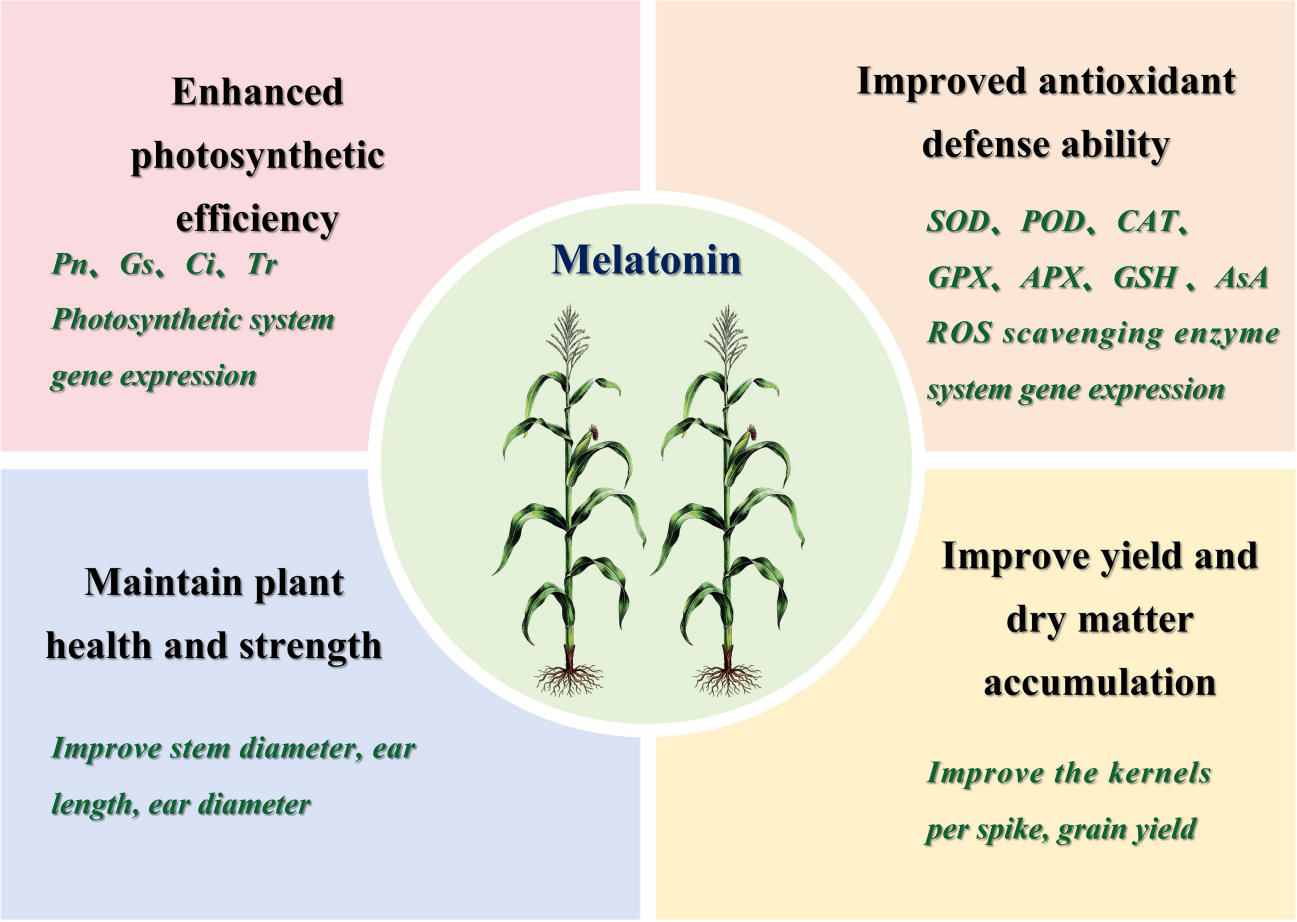
Regulatory mechanism model of exogenous melatonin mediating maize tolerance to post-flowering natural high-temperature stress.

Post-flowering high-temperature stress severely restricts the growth and yield formation of summer maize. Exogenous melatonin application effectively mitigates heat-induced damage by coordinately regulating the photosynthetic system and antioxidant defense system in maize (**Figure 10**). This study confirmed that exogenous melatonin application enhances the photosynthetic capacity of summer maize by upregulating the expression of key photosynthetic genes and increasing the net photosynthetic rate of ear leaves, thereby effectively alleviating post-flowering high-temperature damage. Additionally, melatonin boosts the transcription of genes encoding key antioxidant enzymes, including SOD, POD, CAT, APX and GPX, activates corresponding enzyme activities, and reduces the accumulation of reactive oxygen species (ROS) and malondialdehyde (MDA). The synergistic regulation of the above pathways improves the tolerance of summer maize to post-flowering high-temperature stress. Specifically, maize plants under 150 µmol L^-1^ melatonin (MT150) treatment exhibit better growth status, lower biomass loss, and higher grain yield. Integrated agronomic, physiological and transcriptomic analyses verified that 150 µmol L^-1^ melatonin effectively enhanced the adaptability of summer maize to natural post-flowering high-temperature stress, highlighting the potential of melatonin as a chemical regulator to improve crop heat tolerance. The findings of this study help to elucidate the key mechanisms underlying exogenous melatonin in regulating heat tolerance of summer maize during post-flowering stage, and provide important theoretical guidance for the development of stress resistance, disaster mitigation, yield stability technologies and efficient regulation strategies for maize production.

## Data Availability

The datasets generated and analyzed in this study are available in a public online repository. The repository and corresponding BioProject accession number are listed as follows: Sequence Read Archive (SRA, https://www.ncbi.nlm.nih.gov/sra) under accession number PRJNA1447999.
