# Supplementary figures and images for: Exogenous melatonin alleviates post-flowering natural high-temperature stress in maize (Zea mays L.) by promoting photosynthesis and antioxidant defense responses
Source: Front Plant Sci. 2026 Apr 28;17:1810561. doi: 10.3389/fpls.2026.1810561 (PMC13160915; doi:10.3389/fpls.2026.1810561)

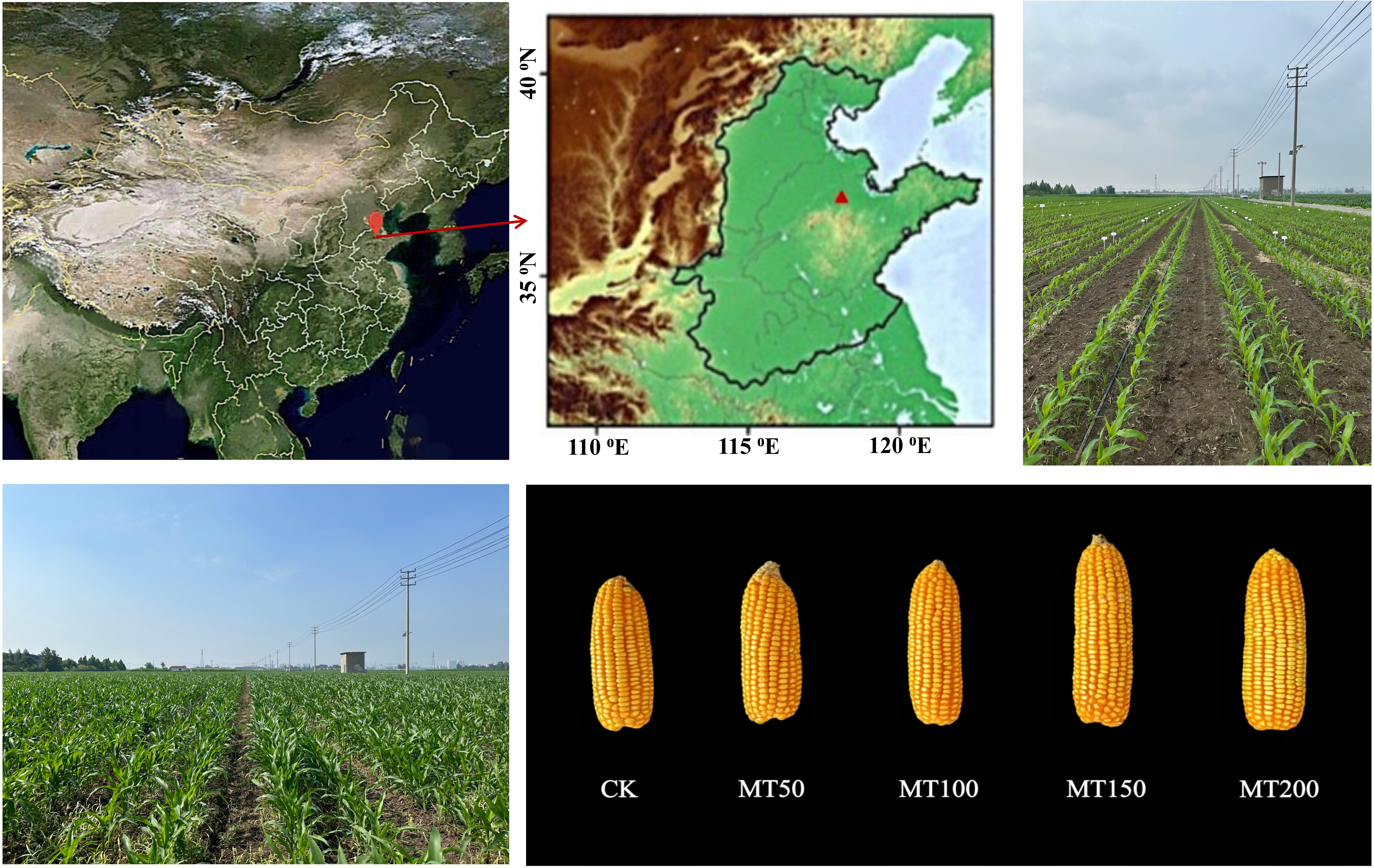

Supplement: Supplementary file 1 [file Image1.jpeg]
